# Pain-related avoidance and endurance behaviour in migraine: an observational study

**DOI:** 10.1186/s10194-019-0962-7

**Published:** 2019-01-18

**Authors:** Ruth Ruscheweyh, Diana Pereira, Monika I. Hasenbring, Andreas Straube

**Affiliations:** 1 Department of Neurology, Ludwig Maximilians University, Marchioninistr. 15, 81377 Munich, Germany; 2 Department of Anesthesiology and Pain Medicine, Ludwig Maximilians University, Munich, Germany; 3 Department of Medical Psychology and Medical Sociology, Faculty of Medicine, Ruhr-University of Bochum, Bochum, Germany

**Keywords:** Migraine, Fear-avoidance, Disability

## Abstract

**Background:**

The role of avoidance and endurance behaviour is well established in chronic musculoskeletal pain, but less is known about its significance in migraine.

**Methods:**

The Avoidance-Endurance Questionnaire behavioural subscales, the Pain Disability Index (PDI), the Migraine Disability Assessment Scale (MIDAS) and the Hospital Anxiety and Depression Scale (HADS) were obtained from 128 migraine patients (90 episodic, 38 chronic). Sixty nine of them were re-evaluated after 3–6 months.

**Results:**

At baseline, there were positive relations between avoidance (especially social avoidance behaviour) and pain-related disability as assessed by the PDI (Wald χ^2^ [1] = 32.301, *p* < 0.001) and the MIDAS (Wald χ^2^ [1] = 14.387, *p* < 0.001). A negative relation of endurance behaviour with PDI scores did not survive multiple regression analysis. In addition, there was a positive relation of social avoidance with the HADS depression score (Wald χ^2^ [1] = 3.938, *p* = 0.047) and a negative relation of endurance (especially the humour-distraction subscale) with the HADS anxiety score (Wald χ^2^ [1] = 6.163, *p* = 0.013). Neither avoidance nor endurance were related to headache intensity or frequency, or to a diagnosis of episodic vs. chronic migraine. 3–6 months after treatment at our headache centre, headache frequency, intensity and pain-related disability were significantly improved (all *p* < 0.01) while avoidance and endurance were unchanged.

**Conclusions:**

This indicates that improvement in headache frequency and disability can be achieved in the absence of changes in avoidance or endurance behaviour. However, because of its significant link to headache-related disability, avoidance behaviour (especially social avoidance) should be investigated as a potential additional target of migraine therapy.

## Background

The fear-avoidance model as described by Vlaeyen [1] is now well established among the psychological mechanisms contributing to the transition from acute to chronic musculoskeletal pain. This model describes how exaggerated fear/anxiety in response to or anticipation of pain (e.g. the fear that physical activity will induce pain) leads to physical and social avoidance behaviour, physical deconditioning and depression, ultimately resulting in more pain and disability. The avoidance-endurance model proposes that in addition to avoidance, pain endurance behaviour in spite of severe pain in the long run also may exacerbate chronic pain, e.g. by leading to continuous physical overload [2]. These models have been extensively studied in musculoskeletal pain, and may also play a role in headache. It has been reported that avoidance and endurance behaviours are frequent in headache [3, 4]. However, they do not seem to be significantly more frequent in chronic headache (headache on ≥15 days/month) compared to episodic headache (headache on < 15 days/month) [3, 4]. Using structural equation modelling in 211 headache patients, a pathway from anxiety sensitivity and headache severity to fear of pain and further on to avoidance and escape behaviour was demonstrated [5]. Another study reported a significant association between anxiety and avoidance behaviour in migraine [6]. Exaggerated avoidance of headache triggers is one special aspect that has been discussed in recent years, and is thought to be at least partly maladaptive [7]. To our knowledge, the relationship of avoidance and endurance behaviour to headache-related disability has not been directly examined, and no longitudinal studies on avoidance and endurance behaviour in headache have been published.

Therefore, in the present study, we investigated (1) if avoidance/endurance behaviour in migraine patients is related to disability and (2) if avoidance/endurance behaviour changes (e.g. in parallel with improvement of migraine) over the course of therapy.

To this end, we used the Avoidance-Endurance Questionnaire (AEQ) to assess avoidance and endurance behaviour in 128 migraine patients at their first presentation at our tertiary headache centre. We assessed the relation of avoidance and endurance with headache frequency, intensity, headache-related disability, depression and anxiety. In addition, we assessed if therapy success after 3–6 months is paralleled by changes in avoidance/endurance behaviour.

## Methods

### Participants

The study was conducted in accordance with the Declaration of Helsinki. It is based on 128 migraine patients who participated in the interdisciplinary outpatient assessment and treatment program in the Upper Bavarian Headache Center at the Department of Neurology, Munich University Hospital, between March 2012 and September 2014. This program is open for patients whose health insurance companies have entered a contract that includes special reimbursement modalities as well as specific follow-up and quality control requirements. Patients came from the entire area of southern Germany, most of them following a direct invitation of their health insurance companies who offered them participation in the program. During their first appointment in the Headache Center, all patients participating in the program completed a set of questionnaires. They also provided written informed consent to use of their data for the quality control that was part of the contract with the health insurance companies, and to publication of the data in anonymized form as part of the quality control process. Consent of the local data protection commissioner was obtained. Criteria for inclusion in the present analysis were: (a) age above 18 years (b) diagnosis of migraine with and/or without aura or chronic migraine according to the International Classification of Headache Disorders (ICHD-3-beta, [8]) and (c) adequate knowledge of the German language. A total of 147 patients were initially recruited, but 19 had to be excluded (1 missing data, 1 age < 18 years, 17 final headache diagnosis different from migraine).

Power analysis indicated that a sample size of 59 would be sufficient to detect a correlation of │Spearman’s rho│ = 0.35 at a power of 0.80 and *p* < 0.05 (two-tailed). Because of the rather lengthy set of questionnaires, we anticipated that only ~ 50% of the patients taking part in the cross-sectional study would also participate in the longitudinal study, so that we aimed at a sample size of 120 patients for the cross-sectional part of the study. For the comparison of chronic with episodic migraine patients, assuming a chronic:episodic ratio of 1:2 in our tertiary care center population, a sample size of 35 chronic and 71 episodic migraine patients was found to be sufficient to detect a group difference at a moderate effect size of Cohen’s d = 0.6 with a power of 0.08 and *p* < 0.05, using a two-tailed Mann-Whitney-U test. Power calculation was performed using G*Power version 3.1.9.2 [9].

### Study design

At their first appointment at the Headache Center, patients filled in a set of questionnaires (specified below) on a tablet PC as part of their initial headache assessment. A headache diagnosis was made by a trained headache physician after taking a detailed headache and medical history and performing a physical examination, according to ICHD-3-beta [8]. In addition, a psychological interview was conducted by a trained psychologist, including: screening for depression and anxiety, also taking into account the results of the Hospital Anxiety and Depression Scale (HADS, see below), the patient’s and his/her family’s history of mental disorders, assessment of life events and daily hassles (including stress at home or workplace), the patient’s and his/her environment’s responses to headache and pain, perceived trigger factors for headache and coping strategies, and further topics as needed. Patients received individual medical and psychological counselling regarding attack treatment, non-pharmacological headache preventive treatment (including advice regarding aerobic exercise and relaxation training, and also addressing individual psychological factors that were detected in the psychological interview and judged to be disadvantageous for migraine patients) and, if indicated, a pharmacological migraine preventive treatment was started according to the guidelines of the German Society of Neurology and the German Migraine and Headache Society (updated version: [10]). Both, the patient and the treating neurologist and/or general practitioner were sent a discharge letter listing all recommendations. Three and six months after their first appointment, patients were contacted and asked to again complete the same set of questionnaires (at home), either by paper and pencil, or by online completion via LimeSurvey (LimeSurvey GmbH, Hamburg, Germany). In the latter case, the patient received an individual pseudonym and password by mail. Consent of the local data protection commissioner was obtained for the use of LimeSurvey. If no answer was obtained, reminders were sent 3 and 6 weeks later. There was no financial compensation for participation.

### Questionnaires

#### Avoidance-Endurance Questionnaire (AEQ), behavioural subscales [2]

The AEQ was developed to assess emotional, cognitive and behavioural fear-avoidance and endurance responses to pain, and has been validated and repeatedly applied in different chronic pain populations [2, 11, 12]. In the present study, only the behavioural subscales were used. These assess behavioural responses to pain within two avoidance subscales, the Avoidance of Social Activities Scale (ASAS, 6 items), the Avoidance of Physical Activities Scale (APAS, 5 items), and two endurance subscales, the Humor/Distraction Scale (HDS, 5 items) and the Pain Persistence Scale (PPS, 7 items). The AEQ was developed from the Kiel Pain Inventory (KPI), and for better comparison with KPI subscales, the two AEQ endurance scales are also reported combined into one Behavioural Endurance Scale (BES, 12 items). Patients indicated for each of the listed behaviours (items) how often they engage in this behaviour on a scale ranging from 0 (never) to 6 (every time), within the past 2 weeks. This was done separately for mild pain and for severe pain. Final subscores were formed by averaging item values within each subscale.

#### Migraine disability assessment scale (MIDAS) [13]

The MIDAS measures the impact of migraine on daily functioning over the past three months. The MIDAS score ranges from 0 to 270 and is derived as the sum of five questions: Number of missed days due to headache at work, in household chores, and in non-work activities, and number of days at work and in household where productivity was reduced by half or more. MIDAS grades are defined as: I (MIDAS score 0–5), II (6–10), III (11–20) and IV (> 20), with grade IV corresponding to the highest disability. Two additional questions assess the number of headache days (MIDAS A) and the average headache intensity (MIDAS B) over the past three months. One more question (not part of the MIDAS but included at this point because of its analogy to MIDAS A) assessed the number of days with acute headache medication within the past three months.

#### Pain disability index (PDI) [14]

The PDI measures the degree to which pain interferes with daily life. Patients rate the pain-related disability in each of 7 areas of daily life (e.g. social activities, household chores) on a scale from 0 (no disability) to 10 (complete disability) in general, a specific time frame is not given. The final PDI score is calculated as the average of these 7 ratings.

#### Hospital anxiety and depression score (HADS) [15]

The HADS assesses anxiety and depression (7 items each, rated on a scale of 0 to 3) in the past week. After reversing those items that indicate higher anxiety/depression by lower scale values, the final HADS-A and HADS-D scores are formed by adding the respective item ratings.

#### Additional questions

Patients were also asked to answer the following questions: “Do you regularly (more than once per week) perform aerobic exercise?”, “Do you regularly (more than once per week) perform relaxation training”, “Do you regularly (daily) take a migraine preventive medication or did you receive Botox® injections for migraine in the past 3 months?”

In addition to what is described above, the set also contained questionnaires on cognitive factors in migraine (results will be published separately) and some mandatory quality control items (e.g. satisfaction with headache management at our centre).

#### Statistics

Statistical analyses were performed using the Statistical Package of Social Sciences (SPSS Statistics; IBM, Ehningen, Germany), version 24 for Windows. Values are mean ± SD unless otherwise indicated. *P* < 0.05 (two-tailed) was considered significant. Because several variables did not show a normal distribution, non-parametric tests were used. Spearman’s rho was used to test for correlations. For qualitative description, correlation coefficients around 0.1, 0.3 and 0.5 were classified as weak, medium and high correlation, respectively [16].

##### Cross-sectional study

For testing the relation between AEQ scores and headache outcome parameters (headache frequency, intensity, frequency of medication intake, PDI and MIDAS scores), and the HADS depression and anxiety scores, zero-order correlations were followed by multiple ordinal regression with those variables showing significant zero-order correlations. Because of high correlations between AEQ-ASAS and AEQ-APAS (see below), only one of the two variables (the one showing the higher zero-order correlation with the respective dependent variable) was included in the regression analysis. The AEQ-BES is a combination of the AEQ-HDS and the AEQ-PPS and was not included in regression analysis. By way of an exploratory analysis, we compared AEQ scores between patients with chronic and episodic migraine using the Mann Whitney U test.

##### Longitudinal study

Baseline parameters (headache frequency, intensity, frequency of medication intake, PDI, MIDAS, AEQ and HADS scores) were compared between patients who participated and those who did not participate in the follow-up using the Mann Whitney U test. Change of these parameters from baseline to follow-up was investigated using Wilcoxon’s test. Difference scores (follow-up minus baseline) were calculated for these parameters and compared between patients who provided follow-up questionnaires at 3 months vs. 6 months using the Mann Whitney U test.

## Results

A total of 128 migraine patients (71 episodic migraine without aura, 19 episodic migraine with aura, 38 chronic migraine) were included (see Table 1 for characteristics).

**Table 1:** Characteristics of the study population (*n* = 128)

Age [years]		37.5 ± 11.8
Sex		116 females (91%)
Episodic migraine [n(%)]		90 (70%)
- without aura [n(%)]		71 (55%)
- with aura [n(%)]		19 (15%)
Chronic migraine [n(%)]		38 (30%)
Migraine history [years]		19.4 ± 12.6
Headache days per month		12.1 ± 7.7
Headache intensity [0–10]		6.7 ± 1.5
Days with intake of acute headache medication per month		8.1 ± 5.6
MIDAS score [0–270]		42.6 ± 39.1
MIDAS grade	I	7 (6%)
	II	13 (10%)
	III	15 (11%)
	IV	93 (73%)
PDI score [0–10]		4.4 ± 2.1
HADS depression score [0–21]		4.7 ± 3.5
HADS anxiety score [0–21]		7.4 ± 3.9

Mean and standard deviation or frequencies are given. *MIDAS* Migraine Disability Assessment Scale, *PDI* Pain Disability Index, *HADS* Hospital Anxiety and Depression scale

### Cross-sectional study

Scores of the AEQ avoidance and endurance behaviour subscales are given in Table 2. The two avoidance scores (AEQ-ASAS and AEQ-APAS) were highly correlated (rho = 0.72, *p* < 0.001) while the correlation between the two endurance scores (AEQ-HDS and AEQ-PPS) was smaller (but still classified as high, rho = 0.47, *p* < 0.001). There were negative correlations between the total endurance score (AEQ-BES) and both avoidance scores (AEQ-ASAS: rho = − 0.46; AEQ-APAS: rho = − 0.48, both *p* < 0.001).

**Table 2:** Avoidance and endurance behaviour scores (AEQ behavioural scores)

	Total (*n* = 128)	Episodic migraine (*n* = 90)	Chronic migraine (*n* = 38)	Statistics (episodic vs. chronic migraine)
AEQ-ASAS (Avoidance of social activity)	2.8 ± 1.2	2.8 ± 1.2	2.7 ± 1.1	Z = − 0.04, *p* = 0.97
AEQ-APAS (Avoidance of physical activity)	3.5 ± 1.0	3.5 ± 0.9	3.4 ± 1.1	Z = − 0.09, *p* = 0.93
AEQ-BES (Behavioural endurance)	2.9 ± 0.8	2.9 ± 0.8	3.1 ± 0.8	Z = − 1.3, *p* = 0.19
AEQ-HDS (Humor/distraction)	2.3 ± 0.9	2.2 ± 0.9	2.5 ± 1.0	Z = − 1.2, *p* = 0.22
AEQ-PPS (Pain persistence)	3.4 ± 1.0	3.3 ± 1.0	3.5 ± 1.0	Z = − 1.1, *p* = 0.28

AEQ subscale scores are given as mean ± standard deviation [0–6]. Note that the HDS and PPS subscales together form the BES subscale, which was included because some of the previous studies list only the BES subscale. Statistics are results of the Mann Whitney U test. *AEQ* Avoidance Endurance Questionnaire

Zero-order correlations between headache outcome parameters (headache frequency, intensity, frequency of acute headache medication intake, headache-related disability as assessed by the MIDAS and PDI) and AEQ scores are given in Table 3. There were medium to high positive correlations between avoidance scores and headache-related disability (PDI and MIDAS scores), and small to medium sized negative correlations between the PDI and AEQ endurance scores (AEQ-BES and AEQ-PPS, Table 3). Multiple regression analysis revealed a significant relation between both, the PDI and the MIDAS scores, and the AEQ-ASAS (Wald χ^2^ [1] = 32.301 and 14.387, both *p* < 0.001) but not with the AEQ endurance scores (*p* > 0.1). Therefore, high avoidance scores were significantly associated with high disability, while there was only a marginal association of high endurance scores with low disability.

**Table 3:** Zero-order correlations between headache characteristics, headache-related disability and AEQ behavioural scores (*n* = 128)

	AEQ-ASAS (Avoidance of social activities)	AEQ-APAS (Avoidance of physical activities)	AEQ-BES (Behavioural endurance)	AEQ-HDS (Humor/ distraction)	AEQ-PPS (Pain persistence)
Headache days per month	− 0.13 *p* = 0.13	−0.15 *p* = 0.093	0.15 *p* = 0.096	0.14 *p* = 0.12	0.11 *p* = 0.20
Headache intensity	0.16 *p* = 0.076	0.09 *p* = 0.30	−0.03 *p* = 0.74	−0.05 *p* = 0.56	−0.02 *p* = 0.86
Days with intake of acute migraine medication per month	− 0.10 *p* = 0.24	−0.12 *p* = 0.20	0.09 *p* = 0.31	−0.01 *p* = 0.94	0.16 *p* = 0.075
MIDAS score	**0.36** ***p*** **< 0.001**	**0.24** ***p*** **= 0.006**	−0.03 *p* = 0.76	−0.06 *p* = 0.53	−0.01 *p* = 0.95
PDI	**0.51** ***p*** **< 0.001**	**0.35** ***p*** **< 0.001**	**−0.19** ***p*** **= 0.028**	−0.16 *p* = 0.066	**−0.20** ***p*** **= 0.026**
HADS-Depression	**0.28** ***p*** **= 0.001**	0.17 *p* = 0.052	**−0.20** ***p*** **= 0.021**	**−0.24** ***p*** **= 0.008**	−0.16 *p* = 0.079
HADS-Anxiety	**0.21** ***p*** **= 0.016**	0.09 *p* = 0.29	−0.12 *p* = 0.18	**−0.26** ***p*** **= 0.003**	−0.006 *p* = 0.95

Spearman’s rho is given. Significant correlations are marked in bold. *AEQ* Avoidance Endurance Questionnaire, *MIDAS* Migraine Disability Assessment Scale, *PDI* Pain Disability Index, *HADS* Hospital Anxiety and Depression Scale

We also tested correlations between AEQ scores and HADS depression and anxiety scores (Table 3). There were small to medium sized positive correlations between the AEQ-ASAS and both HADS scores, and small to medium sized negative correlations of both HADS scores with endurance scores, especially the AEQ-HDS. Multiple regression analysis revealed a significant relation between HADS depression and AEQ-ASAS scores (Wald χ^2^ [1] = 3.938, *p* = 0.047) but not the AEQ-HDS scores (*p* = 0.096). HADS-anxiety scores were significantly related to AEQ-HDS scores (Wald χ^2^ [1] = 6.163, *p* = 0.013) but not to AEQ-ASAS scores (*p* = 0.152).

There were no significant correlations between AEQ scores and headache frequency or intensity, or use of acute headache medication (Table 3). Consistently, there were also no differences in avoidance or endurance scores between episodic and chronic migraine (Table 2; all *p* ≥ 0.19).

### Longitudinal study

All participants were asked to again complete the same set of questionnaires at 3 months and again at 6 months after their first appointment, followed by reminders if no answer was obtained. As the actual date patients filled in the questionnaires varied widely around the two time points (3 months: 14.7 ± 3.1 weeks, range: 9–22 weeks, *n* = 51; 6 months: 29.0 ± 5.5 weeks, range 21–46 weeks, *n* = 44), and only a small number of patients completed both the 3 and the 6 months questionnaires (*n* = 25), we decided to pool data from both time points (including the one which was completed nearer to the intended 3 or 6 month time point, where two completed sets were available). This resulted in 69 patients with available follow-up, corresponding to 54% of the initially included 128 patients (Table 4). Baseline headache characteristics did not differ between patients who participated and those who did not participate in the follow-up (headache frequency: 12.9 ± 7.9 vs. 11.1 ± 7.6 days/month, *p* = 0.14; headache intensity: 6.4 ± 1.6 vs. 7.0 ± 1.3, *p* = 0.077; medication days: 8.7 ± 5.9 vs. 7.3 ± 5.2, *p* = 0.16; MIDAS score: 42.2 ± 39.8 vs. 43.1 ± 38.6, *p* = 0.50; PDI: 4.5 ± 2.1 vs. 4.2 ± 2.1, *p* = 0.48). There were also no significant differences in HADS scores, migraine history, and AEQ scores (data not shown).

**Table 4:** Change in headache outcomes and behavioural parameters at follow-up (3–6 months, *n* = 66–69)

	Baseline	Follow-up (3–6 months)	Statistics
Headache days per month	12.9 ± 7.9	8.5 ± 7.7	**Z = − 5.7, p < 0.001**
Headache intensity [0–10]	6.5 ± 1.6	6.0 ± 1.7	**Z = − 2.6,** ***p*** **= 0.009**
Days with intake of acute migraine medication per month	8.5 ± 5.3	6.3 ± 4.7	**Z = − 3.9, p < 0.001**
MIDAS score [0–270]	42.2 ± 39.8	30.9 ± 39.5	**Z = − 3.0, p = 0.003**
PDI [0–10]	4.5 ± 2.2	3.9 ± 2.4	**Z = - 2.0,** ***p*** **= 0.048**
HADS-Depression	4.9 ± 3.4	4.8 ± 3.3	Z = − 0.5, *p* = 0.65
HADS-Anxiety	7.5 ± 3.9	7.2 ± 3.8	Z = - 0.5, *p* = 0.64
AEQ-ASAS (Avoidance of social activities) [0–6]	2.8 ± 1.2	2.9 ± 1.2	Z = − 0.9, *p* = 0.36
AEQ-APAS (Avoidance of physical activities) [0–6]	3.6 ± 1.0	3.7 ± 1.0	Z = − 1.6, p = 0.12
AEQ-BES (Behavioural endurance) [0–6]	2.9 ± 0.8	2.9 ± 0.9	Z = − 0.3, *p* = 0.73
AEQ-HDS (Humor/distraction) [0–6]	2.2 ± 0.9	2.2 ± 1.0	Z = − 0.7. *p* = 0.51
AEQ-PPS (Pain persistence) [0–6]	3.4 ± 0.9	3.4 ± 0.9	Z = − 0.7, p = 0.51

Values are given as mean and standard deviation. MIDAS, Migraine Disability Assessment Scale; *PDI* Pain Disability Index, *HADS* Hospital Anxiety and Depression scale. *AEQ* Avoidance Endurance Questionnaire. Statistics are results of Wilcoxon’s test. Significant results are marked in bold

Aerobic exercise and relaxation training were recommended to all patients, and a pharmacological migraine preventive treatment was initiated if indicated and consented by the patient. In addition, individual counselling was performed regarding psychological factors that were detected in the psychological interview and judged to be disadvantageous for migraine patients. At follow-up, 57% of the patients indicated to perform regular aerobic exercise (vs. 38% at baseline) and 51% indicated to perform regular relaxation training (20% at baseline). At follow-up, 52% of the patients took migraine preventive medication (24% at baseline).

Statistical analysis revealed a significant improvement in all headache outcome parameters (headache frequency, intensity, acute medication intake frequency, MIDAS and PDI scores) from baseline to follow-up (statistics listed in Table 4). In contrast, avoidance and endurance scores were not significantly changed at follow-up (Table 4). HADS depression and anxiety scores were also not significantly changed from baseline to follow-up (Table 4).

To exclude that results from the 3 and 6 months questionnaires differed significantly, we compared the difference scores (follow-up minus baseline) of the parameters contained in Table 4 between the 3 and 6 months groups (3 months: *n* = 33, 6 months: *n* = 36, all *p* > 0.05, data not shown).

### Discussion

The present study shows positive relations of avoidance, especially social avoidance behaviour, with disability in migraine patients (more avoidance, more disability). Different from avoidance, the relations between endurance and migraine-related disability were negative (more endurance, less disability) and small (not surviving multiple regression). After treatment, headache frequency and disability were significantly improved while avoidance and endurance behaviour were unchanged.

Average AEQ subscores around 3 (on a scale from 0 to 6, see Table 2) confirm previous results that avoidance and endurance behaviour is frequent in migraine patients [3, 4]. It is an interesting question if migraine patients show the same avoidance/endurance pattern as other chronic pain samples. When AEQ scores were exploratively compared with results from the low back pain literature, the present migraine sample had higher social and lower physical avoidance scores [2, 11] and similar or slightly lower endurance scores [2, 11, 12].

In the present study, there were positive correlations of avoidance behaviour with headache-related disability. This is similar to previous results from chronic pain patients [2, 17]. Also consistent with our results showing only marginal negative relations between endurance and pain-related disability, both small negative relations and a lack of correlation between endurance and disability have been reported in other pain disorders [2, 12, 17]. In contrast to results in low back pain patients [2, 12], no correlations between pain intensity and avoidance or endurance emerged in the present study. Headache days/month may be the clinically more important outcome parameter in migraine than pain intensity, but were also not correlated with AEQ scores. Consistently, there also was no difference in avoidance or endurance scores between patients with episodic and chronic migraine. Lack of correlation of avoidance and endurance behaviour with migraine frequency and intensity has been reported before [3]. Only a very large study of ~ 1500 patients found a slightly but significantly increased avoidance behaviour in chronic compared to episodic migraine [18]. In conclusion, avoidance and to a much smaller extent also endurance behaviour seem to be related mainly to headache-related disability, not to headache frequency or intensity in migraine patients. A role of avoidance/endurance behaviour in the transition from episodic to chronic migraine is not supported by the present data.

It has been postulated that pain endurance strategies, typically associated with less disability in the setting of acute or subacute pain, may lead to increased pain and disability in the long run because of continuous overload [2]. Indeed, both avoidance and endurance seem to be associated with worse therapy outcomes in back pain patients [11, 19]. From the present study, there is limited evidence of endurance behaviour being disadvantageous in migraine. In spite of a migraine history of on average 19 years, there was no positive association between endurance behaviour and disability or depression. Endurance behaviour was not more frequent in chronic migraine, suggesting that it is not a risk factor for progression of migraine. It is important to consider that endurance is gradual, ranging from adaptive behaviour to excessive persistence in spite of severe pain [17] and that relations to disability depend on the type of endurance (e.g. excessive endurance vs. task-contingent endurance) [20]. The present results suggest that in migraine patients, endurance behaviour is exerted at rather adaptive levels, not being associated with increased disability or chronicity.

The fear-avoidance model predicts that exaggerated fear/anxiety related to pain leads to avoidance behaviour which will exacerbate pain and disability in various ways, one of them by increasing depressive mood [1]. Exaggerated endurance behaviour has been suggested to relate to positive mood, but on the other hand, it might promote the perception of failure and consequently result in depressive mood [2]. In the present study, higher HADS depression scores were related to higher social avoidance scores and lower endurance scores, although the latter relation did not survive linear regression analysis. These results are similar to previous results in chronic musculoskeletal pain patients, where a positive relation between depression and avoidance is seen, but relations between depression and endurance depend on the type of endurance: positive relations with excessive persistence but negative relations with task-contingent persistence [20]. This corroborates that endurance behaviour in migraine is likely exerted at rather adaptive levels. Higher anxiety scores were only marginally related to higher avoidance scores, but there was a small but significant relation to lower endurance scores on the humor/distraction scale.

In the present study, there were significant improvements in headache frequency, intensity and headache-related disability 3–6 months after treatment in our headache clinic. Remarkably, on average, there was no change in avoidance and endurance scores from baseline to follow-up, suggesting that our interdisciplinary headache treatment does not successfully address these pain-related behaviours. This shows on the one hand that successful reduction of headache-related disability is possible in the absence of changes in avoidance and endurance. On the other hand, because of the marked relation of avoidance to headache-related disability, specifically addressing avoidance, especially social avoidance behaviour, might result in additional benefits on disability. The avoidance-endurance model postulates a bidirectional relationship between these behaviours and pain and disability, predicting that avoidance behaviour is not just the consequence of headache-related disability, but that modification of this behaviour can also have an impact on disability. Maybe patient classification in subgroups on the basis of avoidance, endurance, depression and thought suppression (fear-avoidance, distress-endurance, eustress-endurance and adaptive response subgroups), which predicts treatment success in low back pain [11, 21], could in future help identifying migraine patients that will benefit from interventions addressing avoidance and possibly also endurance behaviour. There was also no change in HADS depression and anxiety scores from baseline to follow-up. This might be due to rather low levels of these scores at baseline (depression: 4.7 ± 3.5, anxiety: 7.4 ± 3.9; the threshold for clinical significance has been suggested to be at > 8 for depression and > 8 or up to > 11 for anxiety [22].

When trying to transfer the avoidance-endurance model from musculoskeletal pain to migraine, some general considerations have to be made. Exacerbation by physical activity during the acute attack is one of the migraine defining criteria [8], so that physical avoidance behaviour is expected to a certain degree. A small part of migraine patients may also experience triggering of migraine attacks by vigorous physical activity (exercise-induced migraine), and therefore avoid exercise also outside the attacks. The fact that aerobic training is an effective migraine preventive treatment [23] suggests that physical avoidance outside the attacks in the long term will be disadvantageous. Regarding social avoidance, during a full-blown attack, many migraine patients need to rest, and tend to cancel social activities. Clinical experience shows that some patients also show a more general social avoidance behaviour, to avoid having to cancel activities at a short notice. The same as in musculoskeletal pain [24], this can lead to social isolation, loss of positive experiences and ultimately depression. The clinical impression is that migraine patients in spite of their headache disorder show high levels of achievement motivation [25, 26], so that endurance behaviour might be expected. Overload of physical structures by endurance, as postulated for musculoskeletal disorders, is unlikely to play a role in migraine. On the other hand, migraine is a highly stress-related disorder [27, 28], so that exaggerated endurance might exacerbate migraine by increasing stress levels. However, no positive relations between endurance and disability or chronicity were found in the present study. In theory, high endurance behaviour might also lead to more frequent intake of acute migraine medication and increased risk of medication overuse headache, which was however also not corroborated by the present data.

Another major difference between musculoskeletal disorders and migraine is that at least in episodic migraine, attacks are separated by pain-free episodes. While the AEQ assesses avoidance and endurance behaviour in the presence of pain, behaviour between the attacks (e.g. social avoidance triggered by fear of the next attack, or stress-enhancing endurance behaviour “I lost time because of my migraine yesterday, I have to make up for that today”) may be also important in migraine. One special aspect of avoidance behaviour between headache attacks is the anxiety and avoidance related to potential headache triggers, which is thought to be at least partially maladaptive, leading to restrictions in lifestyle and impeding habituation to triggers [7], and there is preliminary evidence for a beneficial effect of learning to cope with triggers vs. trigger avoidance [29]. A possible strategy to better assess avoidance and endurance in migraine in future studies would be to reference the behavioural AEQ items to behaviour during and outside headache attacks, instead of during mild and severe pain.

The present study has several limitations. Although baseline characteristics were not different in patients who provided follow-up data and those who didn’t, the relatively low (54%) follow-up participation could nonetheless have caused a bias, e.g. because satisfied patients may be more likely to participate in a follow-up. The relatively low participation also forced us to pool data from the 3 and 6 months time points. The low follow-up participation was probably in part due to the rather lengthy set of questionnaires assessed, and the fact that no financial compensation was provided. In addition, patients tend to make their first appointment in a headache center at a moment where their migraines are at a peak, so that part of the positive outcome might be related to regression to the mean rather than to effect of the therapy. This can only be solved by conducting population-based studies or maybe by randomized studies with a waiting list control. Also, the present patient sample was recruited from a tertiary headache centre, not representing migraine patients from the general population. However, assessing behavioural factors and their relation to headache course may be especially important in these severely affected patients. Further, the validated questionnaires used in the present study use different recall periods, varying from 3 months (MIDAS, number of headache days per month and number of days with acute headache medication per month) over 2 weeks (AEQ), 1 week (HADS) to an unspecified time frame (PDI). This might have artificially reduced correlations between the questionnaires’ results. An additional limitation is that we assessed only the behavioural, not the cognitive subscales of the AEQ. This was done to keep questionnaire filling time within reasonable limits.

## Conclusions

The present study shows that avoidance and endurance behaviour are frequent in migraine, but not modified by our current treatment approaches. Our data demonstrate that improvement in headache frequency and disability can be achieved in the absence of changes in avoidance or endurance behaviour. However, because of the significant relation of avoidance behaviour with headache-related disability, investigating if interventions that specifically target avoidance behaviour further improve the management of migraine would be worthwhile. Future studies should also address avoidance and endurance behaviour between migraine attacks, not only within attacks.

## Abbreviations

AEQ: Avoidance-endurance questionnaire
AEQ-APAS: AEQ, avoidance of physical activities scale
AEQ-ASAS: AEQ, avoidance of social activities scale
AEQ-BES: AEQ, behavioural endurance scale
AEQ-HDS: AEQ, humor/distraction scale
AEQ-PPS: AEQ, pain persistence scale
HADS: Hospital anxiety and depression scale
ICHD: International classification of headache disorders
MIDAS: Migraine disability assessment scale
PDI: Pain disability index
